# Turning the Tide against Herpes Zoster in Rheumatoid Arthritis Patients Treated with JAK Inhibitors

**DOI:** 10.3390/jcm13154423

**Published:** 2024-07-29

**Authors:** Andrea Cito, Marco Fornaro, Angela Carenza, Maria Grazia Anelli, Crescenzio Scioscia, Florenzo Iannone, Giuseppe Lopalco

**Affiliations:** Rheumatology Unit, Department of Precision and Regenerative Medicine and Ionian Area (DiMePre-J), University of Bari Aldo Moro, 70121 Bari, Italy; andreacito.ac@gmail.com (A.C.); marco.fornaro@uniba.it (M.F.); angela.carenza.98@gmail.com (A.C.); mariagrazia.anelli1974@gmail.com (M.G.A.); oetzi72@gmail.com (C.S.); florenzo.iannone@uniba.it (F.I.)

**Keywords:** Janus kinase inhibitor, herpes zoster, safety, vaccine

## Abstract

**Objectives**: This study aimed to evaluate the incidence of Herpes Zoster (HZ) in patients with rheumatoid arthritis (RA) treated with Janus kinase inhibitors (JAKi), and to predict potential risk factors for HZ development. **Methods**: We retrospectively analysed medical records from RA patients at our rheumatology unit who met the 2010 ACR/EULAR criteria for RA and were receiving JAKi. The incidence and course of HZ were assessed through chart review and supplementary phone interviews. **Results**: A total of 198 JAKi-treated patients were monitored for an average of 18.5 months. Nine subjects experienced HZ, resulting in an incidence of 2.95 per 100 patient-years. No demographic or treatment-related differences were found among patients who developed HZ and those who did not. Disease duration (OR: 1.06, 95% CI: 1.01–1.12), time on JAKi treatment (OR: 1.04, 95% CI: 1.009–1.073), higher disease activity at JAKi initiation (OR: 4.16, 95% CI: 1.07–16.17), and at 3-month follow-up (OR: 6.0, 95% CI: 1.35–26.60) were identified as predictors of HZ occurrence. Thirty-six patients received vaccination against HZ, and none reported adverse reactions or flare-ups during a mean follow-up of 9.6 months. **Conclusions**: The incidence of HZ aligns with published data, suggesting that disease and treatment duration, as well as disease activity, are significant predictors of HZ in RA patients on JAKi therapy. Vaccination against HZ proved to be safe and effective, underscoring its potential protective value in this patient population.

## 1. Introduction

Herpes Zoster (HZ) is the clinical manifestation of varicella zoster virus (VZV) reactivation from its latent state in the spinal and cranial sensory ganglia following a primary infection that typically occurs in childhood [1]. It is estimated that almost 30% of the general population will experience HZ in their lifetime [2], with risk increasing with age (over 50 years old) [3], immunosuppression state, or concurrent immunomodulatory treatments [4]. The paraphysiological decline of cell-mediated immunity associated with ageing is one of the main factors explaining the increased incidence of HZ in older adults. Other contributing factors include stress, ongoing immune-regulating therapies, or idiopathic immune-impairing conditions [5]. HZ typically presents as acute pain lasting approximately 2 to 4 weeks, accompanied by characteristic cutaneous manifestations. The skin involvement of HZ generally presents as a painful vesicular rash with a unique unilateral restriction to a single dermatome, although multi-dermatomal presentations are possible. The disease can also disseminate, leading to ocular, vascular, nervous and systemic, despite the availability of anti-viral therapy [6]. The main complication of HZ is post-herpetic neuralgia (PHN), defined as chronic pain with an onset between 1 to 3 months after reactivation and lasting at least 3 to 6 months [7]. Between 20% and 30% of patients are expected to experience this complication, with a prevalence of 0.5 to 1 million [8]. Managing the neuropathic pain resulting from PHN could be extremely challenging due to the peripheral and central nervous system changes induced by the infection. Symptoms such as a sensation of burning, lancinating pain or intense itching along the nerve path are often the patients’ main complaints. Individuals suffering from PHN often experience a decline in their quality of life and psychological well-being. This can lead to potentially long-lasting disability and functional impairment, increasing the total burden on healthcare expenses [8]. Moreover, the economic impact extends beyond direct medical costs to include lost productivity and an increased need for caregiving [9].

Several studies emphasise the rising risk of serious infection among rheumatoid arthritis (RA) patients who receive immunosuppressive treatments [10]. In particular, the incidence rate of HZ in healthy older individuals versus RA patients is estimated to be 0.6 versus 1.5 per 100 person-years (PY) in the 50–60 years age subset and 0.9 versus 1.7 among 61–70 years [11]. These data show a 1.5–2-fold higher risk of HZ occurrence in patients suffering from autoimmune conditions such as RA confronted with the general population [12].

Janus Kinases (JAK) inhibitors (JAKi) are recognized as an effective [13,14,15] and safe [16,17] option for RA management. The JAK family is composed of four intracellular tyrosine kinases: JAK1, JAK2, JAK3 and TYK2 [18]. JAKs bind to the intracellular domain of cytokines and growth factor receptors either as homodimers or heterodimers, thus mediating their signalling pathway. The conformational changes subsequent to the binding of a ligand to its receptor enable JAK activation through auto and trans-phosphorylation. Activated JAKs then phosphorylate other proteins such as signal transducer and activator of transcription (STAT), AKT, MAPK/ERK and phosphoinositide 3-kinase (PI3K), directly involved in regulating the transcription of selected genes [19,20].

JAK2 activity has been mainly linked to erythropoiesis and thrombopoiesis, JAK3 to immune regulation and proliferation of lymphocytes, while TYK2 seems to play a predominant role in mediating anti-viral responses. Gain or lack of function genetic mutations of the JAK pathway are implied in several inflammatory and proliferative diseases, and its blockade resulted in a viable target for their management [21,22,23].

Nevertheless, concerns have been raised regarding the observed increase in HZ incidence among JAKi users [24]. This could be attributed to the protective role of the IFN signalling pathway against viral infections [25,26,27]. Indeed, type I IFN production has been shown to decrease the permeability of the blood–brain barrier (BBB), thereby reducing the likelihood of neurotropic viral infections such as HZ [28]. Conversely, higher levels of type II IFN (gamma) are associated with a selectively reduced specificity of BBB. This allows anti-viral antibodies and other immune cells to migrate from the bloodstream to nervous tissues, enhancing the body’s defence against viral infections [29]. Consistently, VZV has been shown to be able to inhibit IFN alpha and gamma signalling by blocking the STAT1 pathway and upregulating the STAT3 downstream, which leads to virus replication and survival in host tissues [30]. The prominent importance of IFN signalling in driving the host response to varicella zoster virus is also demonstrated by the increasing rates of reactivation observed when its pathway is intentionally inhibited by a monoclonal antibody antagonist of the type 1 interferon receptor (IFNAR), anifrolumab, employed in the management of Systemic lupus erythematosus (SLE) [31].

Therefore, it is believed that the increased rates of HZ among JAKi users may be explained by their mechanism of action, which involves downregulating the JAK/STAT intracellular pathway. Specifically, JAKi hinders IFN signalling by inhibiting the JAK1/TYK2 heterodimer [32]. Murine models lacking TYK2 were found to have reduced IFN production and, consequently, were more susceptible to viral infections. Evaluations in animal models showed a worsening of clinical infection due to STAT3 inhibitions by small-molecule drugs [30]. In vitro inhibition of distinct JAK/STAT-mediated cytokine pathways has been assessed by flow cytometry in peripheral blood mononuclear cells and whole blood samples from RA patients. All JAKis were shown to most potently inhibit the JAK1/TYK2-dependent Interferon (IFN) α signalling pathway mediated by STAT5. Other cytokine maximum inhibitory concentration (IC50) measurements for each JAKi were then normalised to this value to assess potency differences. Very slight differences were found in the inhibition of JAK1-mediated pathways, such as for IL-6 (JAK1/JAK2) and IL-15 (JAK1/JAK3) among all JAKi. On the opposite end, filgotinib demonstrated the greatest JAK2 selectivity, while baricitinib showed the lowest JAK1 selectivity performance [33].

Initial efforts to develop an HZ vaccine led to the commercialization of a live, attenuated VZV formulation capable of boosting cell-mediated immunity to a wide spectrum of selected viral antigens. Although showing promising efficacy rates among older adults against HZ (51%) and post-herpetic neuralgia (67%), vaccination with live attenuated vaccines is contraindicated in individuals who are immunosuppressed or taking immunosuppressive medications due to the risk of possible vaccine-associated reactivation of the disease, even in its disseminated form [34]. Therefore, a preventive approach prior to the start of an immunosuppressive treatment was not yet a viable or safe option.

In contrast, a new non-live recombinant zoster vaccine (RZV) has recently become available for HZ prevention. Glycoprotein E, widely expressed in VZV-infected cells, was the antigen selected for RZV along with the AS01B adjuvant system. This combination proved to greatly enhance IFN serum concentration to levels comparable with those measured during the natural infection. The data demonstrating its efficacy [35,36] and safety [37] in adults over 50 years old have broadened its use, including in patients undergoing immunosuppressive treatments who could not be safely vaccinated with the previously available attenuated vaccine. This expanded applicability has provided a crucial option for those until that moment at risk of complications from live vaccines. The aim of our retrospective study was to assess HZ incidence in a cohort of RA patients treated with JAKi in a real-life setting. We sought to identify possible risk factors and additionally explore the beneficial role of the RZV in the daily clinical management of RA patients, potentially leading to improved patient outcomes and reduced healthcare burden associated with HZ.

## 2. Materials and Methods

We performed a retrospective study using data collected from our electronic health records from 2017 to 2023. Our focus was on patients meeting the diagnostic criteria for rheumatoid arthritis (RA) as defined by the 2010 ACR/EULAR classification criteria for RA [38] who started treatment with a JAK inhibitor at our rheumatology unit in Bari. Retrieved data included patient demographics, clinical profiles, and previous therapeutic interventions. Disease activity was assessed trough the Clinical Disease Activity Index (CDAI) validated score both at baseline and at the 3-month follow-up. Information on ongoing medications was concurrently gathered. Cases of HZ reactivation were investigated by reviewing medical charts, and when necessary, supplementary information was obtained through direct telephone correspondence. HZ cases were defined by the presence of a typical unilateral dermatomal vesicular rash, with diagnosis established as the primary determination by the treating physician. Moreover, all causes of JAKi discontinuation were recorded. Within our cohort, 36 RA patients received RZV according to the recommended schedule of two injections administered within a 60-day period. After the second vaccine dose, these patients were excluded from the principal cohort and followed separately. Data, including information on potential side effects and reactivations, were collected to assess the effectiveness and safety profile of the RZV. Written informed consent was obtained from each enrolled patient. The study was conducted in accordance with the ethical guidelines of the Declaration of Helsinki and received approval from the Ethics Review Board of the Policlinico of Bari (protocol n° 5277), with the ethical approval date being 7 June 2017. The Kolmogorov–Smirnov test was used to evaluate the distribution of continuous variables. Demographics and disease characteristics were evaluated using standard descriptive statistics. Categorical variables were presented as numbers or percentages, and continuous variables as either mean and standard deviation (SD) or median with interquartile range (IQR). Statistical analysis was performed using SPSS IBM Software (Version 21.0, Armonk, NY, USA).

## 3. Results

In our study, we enrolled 198 RA patients starting a JAKi treatment. The majority of patients are female, with 88.9% in the HZ reactivation group and 85.7% in the no-HZ reactivation group. The average age of patients who experienced HZ reactivation is 58.7 years (±16.7), while the average age for those who did not have reactivation is slightly lower at 54.1 years (±13). Patients who experienced HZ reactivation have a longer average disease duration from onset to first JAKi introduction of 19.8 months (±5.9) compared to those without reactivation, who have an average duration of 11.5 months (±4.7). The follow-up period on JAKi was also longer for the HZ group at 34.2 months (±22.7) versus 17.8 months (±17.2) for the non-HZ group. Usage of anti-TNF therapies varies, with 55.6% of the HZ group having no previous anti-TNF agent, compared to 44.4% in the non-HZ group. A substantial portion of each group had previously used other bDMARDs: 44.4% in the HZ group versus 53.4% in the non-HZ group. Among JAKi, baricitinib was notably predominant in the HZ group, used by 77.8% of patients, while its use was significantly lower in the non-HZ group at 48.7%. More in detail, in the whole cohort comprising HZ and non-HZ patients, 99 were on baricitinib, 43 on upadacitinib, 39 on filgotinib, and 17 on tofacitinib. A high prevalence of baseline oral glucocorticoid use was noted in both groups, with 77.8% in the HZ group and 72% in the non-HZ group. The overall population was observed for an average duration of 18.5 (±17.8) months. Globally, 84 JAKi discontinuations were recorded: 21 due to primary ineffectiveness, 42 due to loss of efficacy, 13 due to adverse events (seven infections, including four pulmonary infections, two zoster reactivations, and one recurrent urinary infection; four drug-related adverse events; and one constitutional symptom causing drug discontinuation), 2 due to remission, and 6 for unknown reasons. During the follow-up period, nine patients experienced HZ reactivation. The overall incidence of HZ in our cohort was 2.95 cases/100 patients per year. The average recorded time from the onset of JAKi treatment to HZ reactivation was 23.5 (±5.6) months. Among these cases, seven were on baricitinib, one on upadacitinib, and one on tofacitinib. No HZ reactivations were recorded in patients undergoing filgotinib treatment. All patients who experienced HZ presented with mild symptoms, ranging from transient skin manifestations to self-limiting neuropathic pain. Two of them (22.2%) required permanent discontinuation of the drug, four (44.4%) underwent a temporary suspension, and for the remaining three (33.3%), no action was taken as these patients reported the infection only after its resolution. The characteristics of the nine patients who suffered from HZ are depicted in Table 1.

Regardless of the decision made, 100% of the patients who experienced HZ reactivation achieved complete resolution. None of the patients suffered from chronic neurologic sequelae in the form of post-herpetic neuralgia. Table 2 compares the demographic and clinical characteristics of RA patients who experienced HZ reactivation with those who did not. No significant statistical differences in demographic characteristics were observed between the groups. Similarly, there were no differences in the disability index and co-treatments at the time of the first JAKi prescription. Patients who experienced HZ reactivation were found to have a significantly longer disease duration before JAKi prescription (19.8 ± 16.2 months vs. 11.5 ± 9.1 months, *p* < 0.01). Furthermore, the duration of JAKi treatment was significantly longer for patients who experienced HZ reactivation compared to those in the other group (34.2 ± 22.7 months vs. 17.8 ± 17.3 months, *p* < 0.01). Of note, a higher percentage of patients exhibiting high disease activity according to the CDAI score (>22) was observed in the group that experienced HZ reactivation, both at baseline (55.6% for HZ vs. 22.8% for no-HZ, *p* < 0.05) and at 3-month follow-up (33.3% for HZ vs. 7.4% for no-HZ, *p* < 0.05). Finally, a multivariate logistic regression model, adjusted for age, sex, disease duration, time on JAK inhibitor (JAKi) treatment, CDAI both at baseline and 3-month follow-up, baseline conventional synthetic DMARDs, baseline steroid treatment, and the type of JAKi, revealed that the main independent predictors of HZ reactivation were: disease duration (OR: 1.065, 95% CI: 1.011–1.123, *p* < 0.05), time on JAKi treatment (OR: 1.040, 95% CI: 1.009–1.073, *p* < 0.05), and higher disease activity (HDA) assessed before starting JAKi treatment (OR: 4.16, 95% CI: 1.07–16.17) and at 3-month follow-up (OR: 6.0, 95% CI: 1.35–26.60). Thirty-six RA patients (16.3%) received the RZV vaccine. Of these patients, 28 (77.8%) were female, with a mean age of 57 ± 13 years. All of these patients received the vaccine after a mean duration of 15 ± 14 months from the start of JAKi therapy. Only two patients received the first dose of the vaccine before starting JAKi therapy. Fourteen were treated with baricitinib, 8 with tofacitinib, 7 with filgotinib, and 7 with upadacitinib. None of them experienced HZ reactivation or subsequent adverse reactions during the mean follow-up period of 9.6 ± 8.3 months.

## 4. Discussion

In our study involving RA patients treated with JAKi, we observed an overall HZ incidence of 2.95 cases per 100 patient years. Previous studies on this topic are summarised in a 2019 meta-analysis that included a total of 11,144 patients undergoing JAKi treatment (5888 with tofacitinib, 3520 baricitinib and 1736 with upadacitinib). The rate of HZ recurrence across all treatments was 3.23 cases per 100 patient-years, with only baricitinib showing a statistically significant increase in incidence rates: 2.86 (95% CI: 1.26, 6.50) vs. placebo [39]. These results are comparable with those found in our work. Among the reactivation cases, seven were recorded in patients taking baricitinib, 1 case occurred in patients treated with upadacitinib, and another one in patients on tofacitinib. No HZ reactivation was documented in patients undergoing therapy with filgotinib. These results are consistent with findings from large safety analyses, which showed higher HZ incidence rates with baricitinib and tofacitinib at 4.4 and 3.6/100 patients-year, respectively [40,41]. The susceptibility to HZ in patients receiving JAKi treatment and the differences in rates among the drugs administered in our cohort likely stem from subtle differences in their molecular interactions with JAK receptors and STAT proteins. Cellular assays have shown that all JAKis effectively suppress JAK-1-dependent IL-6 and IFNα secretion, with similar average inhibition capacities [33]. This ability to inhibit pathways of crucial inflammation-driving cytokines such as IL-6 underlies the efficacy of JAKi in managing RA. However, reducing IFNα signalling may weaken immune functions, increasing the risk of infections.

It is important to note that considering only JAK1 inhibition to explain all effects and adverse events of JAKi may be misleading, as JAK receptors pair to signal downstream. JAK2-dependent cytokine pathways are less affected by JAKi. For instance, filgotinib, upadacitinib and baricitinib show more than threefold less inhibitory power on the JAK1/JAK3 axis compared to JAK1-dependent IFNα pathways, with filgotinib demonstrating the greatest selectivity by showing almost seven-fold lesser activity on IFNγ/pSTAT1 production through JAK1/JAK2. Moreover, filgotinib showed less impairment on JAK2/TYK2 and JAK2/JAK2-dependent cytokines such as IL-12, IL-23, and Granulocyte-Macrophage colony-stimulating factor (GM-CSF). The resulting lower inhibition of these cytokines in neutrophils and other granulocytes translates into better preserved antipathogenic function and, therefore, a better capacity to fight pathogens. Conversely, baricitinib showed the worst JAK1 selectivity (≤5.1-fold for JAK1 versus non-JAK1 pathways) [33].

Further insights into the interplay between JAK1 selectivity and infection risk are provided by real-life data on the employment of a novel JAK2-selective drug, fedratinib, used in the therapy of intermediate or high-risk, primary or secondary myelofibrosis. Safety data for fedratinib show very low rates of HZ reactivation, similar to those occurring with filgotinib [42].

Additionally, we identified disease duration and time on JAKi therapy as major predictive factors for HZ infection. The cumulative dose of JAK inhibitors may be a potential cause of HZ reactivation in RA patients, underscoring the importance of vaccination to reduce this risk in RA patients undergoing long-term treatment. We also found that the level of disease activity assessed by CDAI score at the time of the first introduction of JAKi therapy positively correlated with HZ reactivation rates, regardless of prior disease duration. Notably, patients who initiated JAKi treatment but did not achieve a sufficient response and, thus, remained in HDA state at the 3-month follow-up exhibited a higher rate of HZ infection compared to patients who achieved remission. These results may be explained by the natural course of RA, which is burdened by a higher incidence of infection events. Among other risk factors, disease activity is widely recognized to correlate with an increased rate of infections, irrespective of disease duration. The strong association between disease activity levels and the risk of HZ reactivation underscores the complex relationship between the immune dysregulation underlying RA pathogenesis and the increased susceptibility to infections among these patients. High disease activity not only reflects ongoing inflammation but also suggests potential disruptions in immune homeostasis, predisposing patients to viral reactivation. An analysis of the RADIUS1 cohort investigated the tight relationship between disease activity (assessed through CDAI), reduction in quality of life reduction (measured with the Health Assessment Questionnaire Disability Index [HAQ-DI]) [43] and the prevalence of major infectious events in RA. Patients with mild, moderate and severe disease activity were found to have infection rates that were 2.7, 4.3 and 4.8-fold higher, respectively, than those in RA patients in remission. Similarly, an increase in HAQ-DI of 0.4 units significantly correlated with a higher occurrence of infection among RA patients [44]. Therefore, achieving and maintaining disease remission or low disease activity should be a primary goal in RA management to mitigate infection risks, including HZ.

Secondly, inadequate control of disease activity often leads to increased use of immunosuppressive medications, such as steroids, which are recognized as potential risk factors for infections [45,46,47].

In particular, as emerged from the CORRONA registry, a daily prednisone dose of 7.5 mg or higher led to an increased rate of HZ occurrence in RA patients (HR 1.78) compared with non-glucocorticoid users [48]. This includes an association with disseminated VZ infection in RA patients [49].

Our study confirmed that JAKi could be associated with HZ, with an overall incidence in line with findings previously reported in the literature [23,35,36,37,39]. While this outcome might be viewed by clinicians as a major drawback in therapeutic choices, it should be noted that all cases of HZ reactivation recorded in our study had favourable outcomes. There were no severe cases, and no chronic sequelae such as PHN were observed. Almost a third of the patients did not halt JAKi treatment (mainly because they achieved complete resolution before being able to consult with us), and among those who were advised to suspend treatment, only a minority (22%) needed permanent suspension, which was nonetheless followed by complete resolution of HZ symptoms. This offers reassurance about the safety profile of JAKi, even in the case of an HZ reactivation. Another key recognition is that, due to their effectiveness and quick onset of action, JAKi can ensure prompt and sustained disease remission [50], thereby reducing the overall reactivation rate associated with disease activity [51,52]. Furthermore, achieving early and stable control of the disease reduces the need for immunosuppressive medication, another clear risk factor for infections. Being able to assess an estimated risk of infection before starting an immunosuppressive treatment would be a significant step forward in achieving a patient-tailored decision-making process. Several risk scores have been proposed for this purpose, mainly accounting for variables such as age, a number of concomitant comorbidities, previous personal history of serious infections and use of immune-modulating medications. A specific RA-centred score, the Rheumatoid Arthritis Observation of Biologic Therapy (RABBIT), has been recently developed in the form of an online calculator (https://rheumcalc.com/rabbit-infection-score/ accessed on 7 June 2024) that could help in stratifying the risk of infection at one year [53]. However, currently, no scoring system has reached validation. The lack of a reliable pre-therapeutic stratifying system for infectious risk advocates for the need for a preventive approach. Our findings, in line with a recent prospective observational study in a cohort of RA patients on biological or JAKi treatment, confirmed the safety and effectiveness of the RZV in preventing HZ in patients starting JAKi therapy [54]. Furthermore, the study investigated the possible effects of ongoing JAKi treatment on RZV immunogenicity at the time of inoculation. For this purpose, anti-VZV IgG serum levels of patients who received RZV while undergoing bDMARD or JAKi medications were collected and compared with those of healthy controls (HC). A similar magnitude in anti-VZV IgG titres was observed in both groups after each dose, with no statistical difference compared to HC receiving RZV as part of routine vaccinations. These results underline that the immunogenic power of RZV is not affected by ongoing JAKi or bDMARD medications [54]. Among all the patients in our cohort, 36 followed our recommendation to receive the RZV in accordance with the EULAR guidelines for adults with autoimmune rheumatic diseases. No one reported symptoms or signs of clinical reactivation of HZ or experienced significant side effects from the vaccination. This further confirms the already available data on the effectiveness and safety of the RZV.

Thus, the systematic use of anti-herpes zoster vaccination in patients requiring immunosuppressive medications emerges as a valuable preventive strategy against infection.

Broader public health implications could be inferred from our study, particularly regarding vaccination strategies for immunocompromised populations. Immunisation against preventable infections such as HZ is paramount in reducing disease burden and healthcare costs associated with complications [55]. Efforts to increase awareness among healthcare providers and patients about the importance of vaccination, including the availability of newer, more efficacious vaccines like RZV, are essential for improving vaccine uptake rates and protecting vulnerable populations.

One limitation of our study is its small sample size. Addressing this issue could mean including additional diagnoses for which JAKi are indicated, such as psoriatic arthritis and ankylosing spondylitis. Additionally, considering the cumulative dose of oral steroids and other immunosuppressive medications administered during the follow-up period could be important for a more accurate assessment of reactivation risk. Further research is warranted to address the remaining questions and gaps in knowledge identified by the study. Large-scale prospective studies with wider patient populations and longer follow-up periods are needed. These studies might confirm the generalizability of our findings and elucidate additional risk factors for HZ reactivation in RA patients. Additionally, they will assist in developing a reliable and validated score to stratify a patient’s infection risk. Moreover, investigations into the immunological mechanisms underlying JAKi-associated HZ reactivation may provide insights into potential preventive strategies and therapeutic interventions. Furthermore, insights into the effects of JAKi on humoral response and the molecular mechanisms explaining the non-impairing effect on RZV immunogenicity are required to provide a comprehensive understanding of the matter. Finally, due to the small sample size and different prescription times, we cannot clearly define a different risk for the four JAKi currently marketed in Europe.

## 5. Conclusions

Our study highlights the essential need for vigilant monitoring for HZ and proactive vaccination strategies in patients with RA, especially those beginning treatment with JAKi. The finding that both disease duration and activity level are significant risk factors for HZ reactivation underscores the critical importance of sustained disease management. Effective control of RA not only improves symptoms but also significantly lowers the risk of viral reactivation, enhancing overall patient well-being.

Furthermore, our study corroborates previous evidence that suggests a higher occurrence of HZ among patients treated with JAKi. Notably, the incidence rates vary across different inhibitors, with baricitinib and tofacitinib associated with higher rates of reactivation compared to upadacitinib and filgotinib. This variability highlights the need for personalised treatment plans that consider the differential risk profiles of each JAKi, enabling clinicians to tailor therapy based on individual patient risk factors for infections.

In addition to what is currently in literature [39], our study not only confirmed the overall HZ incidence in patients undergoing JAKi treatment but also compared those who were administered RZV with those who were not and identified positive reactivation-predicting factors.

Our findings strongly support the integration of anti-herpes zoster vaccination into the standard management protocol for RA, particularly both prior to the initiation and while on JAKi therapy. The absence of an impairing effect on RZV immunogenicity when administered to patients already under JAKi medications [54], along with the safety derived from the new non-live recombinant formulation that does not carry the reactivation risk associated with live-attenuated vaccination, supports the confident implementation of VZV vaccination at any point during JAKi treatment. This preventive strategy not only safeguards against the direct impacts of HZ but also mitigates broader healthcare implications by reducing both direct and indirect costs associated with the disease. It represents a critical step towards minimising the overall burden of illness in immunocompromised populations.

In a broader context, our research reinforces the intricate link between immunomodulatory therapy, disease activity, and the enhanced risk of viral infections within the RA patient population. It confirms the protective benefits of vaccination and highlights the need for a more refined, patient-centred approach to disease management and infection prevention. This strategy should encompass a comprehensive evaluation of patient-specific risk factors and preferences to ensure optimal therapeutic outcomes.

Critical to this patient-focused approach is the concept of shared decision-making, which involves a collaborative process where healthcare providers and patients discuss available treatments to decide together on the best course of action. This process is fundamental, given the complex interplay between treatment efficacy, safety profiles, and infection risks associated with JAKi.

By integrating patient preferences, values, and perceptions of risk into the decision-making process, clinicians can enhance treatment outcomes and boost patient satisfaction. Furthermore, providing detailed education and counselling about the risks and benefits of different therapeutic options, including vaccination strategies, empowers patients to make well-informed decisions that align with their personal health goals.

Implementing these strategies can substantially reduce the incidence of HZ and its complications, thereby improving the quality of life and clinical outcomes for RA patients. As we continue to explore the nuances of JAKi treatment and its implications, our findings underscore the need for ongoing research and adaptation of treatment protocols to better meet the necessities of this exposed patient group. This comprehensive approach not only addresses immediate health concerns but also contributes to the broader public health goal of reducing the burden of infectious diseases in immunocompromised populations.

## Figures and Tables

**Table 1:** Characteristics of rheumatoid arthritis patients who experienced HZ infection on JAK inhibitors.

N. Patient	Sex	Age	Disease Duration (Months)	JAKi	csDMARD	Steroid (PDNeq) mg/die	Time since JAKi Introduction (Months)	Kind of Reactivation (Localisation)	Action Taken	Outcome
1	F	80	10	Baricitinib	MTX	5	18	UNK	Temporary suspension	Complete resolution
2	M	49	11	Baricitinib	/	2.5	27	Mono-dermatomeric (left shoulder)	No suspension	Complete resolution
3	F	29	27	Tofacitinib	/	5	3	Mono-dermatomeric (Retroauricular)	Temporary suspension	Complete resolution
4	F	82	27	Baricitinib	/	20	36	Mono-dermatomeric (Sub-mammary)	Temporary suspension	Complete resolution
5	F	57	15	Baricitinib	/	5	12	UNK	Temporary suspension	Complete resolution
6	F	66	20	Baricitinib	MTX	0	45	Mono-dermatomeric (Right shoulder)	No suspension	Complete resolution
7	F	51	7	Baricitinib	/	5	45	Mono-dermatomeric (Upper lip)	Temporary suspension	Complete resolution
8	F	65	57	Baricitinib	MTX	5	8	Mono-dermatomeric (Abdomen)	No suspension	Complete resolution
9	F	49	4	Upadacitinib	/	0	18	Mono-dermatomeric (Right arm)	Permanent Suspension	Complete resolution

Abbreviations: csDMARD: conventional synthetic disease-modifying antirheumatic drug; HZ: herpes zoster; PDNeq: prednisone equivalent; UNK: unknown data; JAK: Janus Kinase.

**Table 2:** Comparison of demographic, clinical, and therapeutic features between RA patients with and without HZ reactivation.

Variables	HZ Reactivation (n. 9 pt)	No-HZ Reactivation (n. 189)
Age (SD)	58.7 (16.7)	54.1 (13)
Female (%)	8 (88.9)	162 (85.7)
Disease duration (SD) from onset to JAKi introduction, months	19.8 (5.9)	11.5 (4.7) *
Follow-up on JAKi (SD), months	34.2 (22.7)	17.8 (17.2) **
Diabetes (%)	0 (0)	12 (6.3)
RF/ACPA+ (%)	7 (77.8)	145 (76.7)
Previous anti-TNFi (%)		
none	5 (55.6)	84 (44.4)
1	3 (33.3)	54 (28.6)
2	1 (11.1)	51 (27.0)
Previous RTX(%)	2 (22.2)	28 (14.8)
Previous other bDMARD(%)	4 (44.4)	101 (53.4)
JAKi type(%)		
baricitinib	7 (77.8)	92 (48.7)
tofacitinib	1 (11.1)	16 (8.5)
upadacitinib	1 (11.1)	42 (22.2)
Methotrexate/leflunomide(%)	4 (44.4)	61 (32.3)
Baseline oral glucocorticoid (%)	7 (77.8)	136 (72.0)
Baseline oral glucocorticoid dose (PDNeq) mg/die (SD)	5.3 (5.9)	4.9 (4.7)
HAQ-DI (SD)	1.5 (0.6)	1.4 (0.8)
Baseline CDAI (SD)	20.7 (8.9)	17.6 (10.3)
CDAI High disease activity at baseline (%)	5 (55.6)	43 (22.8) **
CDAI High disease activity at 3-months (%)	3 (33.3)	14 (7.4) **

Abbreviations: ACPA: Anti-Citrullinated Protein Antibody; CDAI: clinical disease activity index; bDMARD: biologic disease-modifying antirheumatic drug; HAQ-DI: Health Assessment Questionnaire Disability Index; HZ: herpes zoster; PDNeq: prednisone equivalent; JAK: Janus Kinase; RF: rheumatoid factor; RTX: rituximab; SD: standard deviation; TNFi: tumour necrosis factor inhibitors. * *p* < 0.05, ** *p* < 0.01 vs. HZ reactivation.

## Data Availability

The data that support the findings of this study are available from the corresponding author upon reasonable request.ors declare no conflicts of interest.
